# Inkjet Printing of GAP/NC/DNTF Based Microscale Booster with High Strength for PyroMEMS

**DOI:** 10.3390/mi11040415

**Published:** 2020-04-14

**Authors:** Yining He, Xiuti Guo, Yanling Long, Guangwu Huang, Xiangpu Ren, Chuanhao Xu, Chongwei An

**Affiliations:** 1 School of Environment and Safety Engineering, North University of China, Taiyuan 030051, China; heyining1009@163.com (Y.H.); ironpan3160899@gmail.com (X.G.); longyanling163@163.com (Y.L.); kuankuanme@163.com (G.H.); Renxiangpu@163.com (X.R.); 18434363821@126.com (C.X.); 2 Shanxi Engineering Technology Research Center for Ultrafine Powder, North University of China, Taiyuan 030051, China

**Keywords:** inkjet printing, 3,4-dinitrofurazanofuroxan (DNTF), microscale booster, high strength, high density

## Abstract

In order to improve the mechanical strength of micro-booster based on 3,4-dinitrofurazanofuroxan (DNTF), 2,4-toluene diisocyanate (TDI) was introduced into the composite binder of nitrocotton (NC) and glycidyl azide polymer (GAP). A full-liquid explosive ink containing DNTF, binder and solvent was printed layer by layer. By the polymer cross-linking technology, the inkjet printed sample with three-dimensional network structure was obtained. The morphology, crystal form, density, mechanical strength, thermal decomposition and micro scale detonation properties of the printed samples were tested and analyzed. The results show that the printed sample has a smooth surface and a dense internal microstructure, and the thickness of the single layer printing is less than 10 μm. Compared with the raw material DNTF, the thermal decomposition temperature and activation energy of the printed samples do not change significantly, indicating better thermal stability. The addition of curing agent TDI increases the mechanical properties and charge density of the energetic composites. The elastic modulus and hardness are increased by more than 20%. The charge density can attain 1.773 g·cm^−3^, which can reach 95.5% of the theoretical density. The critical detonation size of the sample can reach 1 mm × 0.01 mm or less and the detonation velocity can achieve 8686 m·s^−1^, which exhibits excellent micro-scale detonation ability.

## 1. Introduction

Based on advanced manufacturing and integration ideas, microelectronic systems, machinery systems and chemical energy systems are integrated into micro electro-mechanical systems (MEMS) with initiating function, which are also known as MEMS pyrotechnics. Using MEMS pyrotechnics technology, ignition or detonation can rely on an energetic chip with centimeter or millimeter size, which can significantly promote the miniaturization, light weight and intelligence of the weapon system [1,2,3,4,5,6]. Recently, MEMS initiating device technology has developed rapidly and has been used in micro propulsion and micro initiators [7,8,9,10,11,12,13,14,15].

The MEMS detonation train is a typical application of MEMS pyrotechnics in ammunition. It is mainly composed of micro energy transformer, micro initiating charge, MEMS charge slider and flyer subassembly, micro-scale booster explosive and so on. Micro-scale booster explosive plays an important role in the MEMS detonation train, which directly determines the safety and reliability of the detonation train. Therefore, the micro-scale charge technology with high quality and high precision is a research focus on the field of MEMS initiator [16,17]. In 2005, Dr. B. Fuchs et al. [18] developed an explosive ink formulation called EDF-11, which was composed of polyvinyl alcohol (PVA), ethyl cellulose (EC), water, alcohol and hexanitrohexaazaisowurtzitane (CL-20) particles. The explosive ink was loaded into the cavity of the MEMS substrate by direct writing technology. Although the explosive ink had more than 72% solid, it could be written fluently. After the ink was cured, the detonation critical size and detonation velocity can reach 86 μm (0.51 mm charge width) and 7150 m·s^−1^, respectively. Subsequently, Z. Q. Zhu et al. [19], D. J. Wang et al. [20], Q. B. Li et al. [21] designed various CL-20-based explosive ink formulations and respectively used direct writing technology to write them in micro grooves. The minimum detonation critical size could reach 0.17 mm (1 mm charge width) and the detonation velocity was up to 8000 m·s^−1^.

In 2011, Andrew C. Ihnen et al. [22,23,24] opened a new path to introduce ink-jet micro manufacturing technology into printed micro nano structure energetic materials. The hexogen (RDX) and pentaerythrite tetranitrate (PETN) particle-free explosive inks were prepared and printed by DMP-2800 inkjet printer and the inkjet printing mechanism of explosive ink was studied initially. Since 2017, C. W. An et al. [25,26,27,28] systematically researched the inkjet printing micro-charged technology for MEMS pyrotechnic devices. CL-20, 3,4-dinitrofurazanofuroxan (DNTF) and PETN based explosive inks were developed and charged in the micro grooves by inkjet printing process. Moreover, the detonation ability of inkjet printing samples at sub-millimeter scale were investigated. Among them, the DNTF/glycidyl azide polymer (GAP)/EC based printed sample has a density of 90% theoretical maximum density (TMD) or more, a critical detonation size of 1 × 0.01 mm, and a detonation velocity of 8500 m·s^−1^, showing good molding effect and excellent detonation ability at micro scale. However, the micro charge, including the GAP/cellulose binding system, has a relatively poor mechanical properties and it was easily damaged by impact forces, which limited its application in MEMS detonation train. Based on the previous research work of our research team, an appropriate amount of curing agent i.e., 2,4-toluene diisocyanate (TDI) was introduced into the explosive ink formulation based on GAP/cellulose bonding system. Using the reaction between the hydroxyl groups in the GAP and the isocyanate in the TDI molecule, the energetic composites with three-dimensional grid structure were obtained. In addition, the molding effect, microstructure, thermal decomposition performance and detonation ability of printed samples were also tested and analyzed.

## 2. Experimental Section

### 2.1. Materials

In this study, all-liquid explosive ink was prepared by dissolving DNTF (200 μm, produced by Gansu Yinguang Chemical Industry Group Co., LTD., Baiyin, China), nitrocotten (NC, supplied by Sichuan Northern Nitrocellulose Co., Ltd., Luzhou, China) and GAP (purchased from Liming Chemical Research Institute, Luoyang, China) into acetone (AR, provided by Tianjin Shentai Chemical Reagent Co., Ltd., Tianjin, China), as well as TDI (obtained from Tianjin Dengke Chemical Reagent Co., Ltd., Tianjin, China) and dibutyltin dilaurate (T-12, produced by Beijing Chemical Plant; Pure, Tianjin Shentai Chemical Reagent Co., Ltd., Tianjin, China).

### 2.2. Design and Preparation of Explosive Ink

Xu et al. [29] designed many explosive ink formulations based on GAP/DNTF. They found when the content of the main explosive is about 90%, the printed sample had good detonation performance and molding effect. Thus, the ratio of main explosive (DNTF) to binder of explosive ink in this paper was remained at 9:1. However, the component of bonding system was optimized to meet the high precision and high strength of printing materials. Firstly, the binder system should convert into the solid state instantaneously after solvent evaporation. After repeated experiments, if the mass ratio of GAP/NC was higher than 8:2, the composites could not become solid. That is to say, the maximum ratio of gap to NC is 8:2. Secondly, the molar ratio of reaction groups (i.e., the molar ratio of isocyanate to hydroxyl group) should be equal to 1:1. Based on this principle, the mass ratio of GAP/NC to TDI was calculated as 21:1. Finally, according to the solubility of each component, acetone was selected as the solvent of the explosive ink and the acetone content was determined as 83.24 wt.%. In summary, the explosive ink formulation (denoted as GNT ink) can be confirmed and tabled in Table 1. As a contrast, an explosive ink formulation without the curing agent (marked as GN ink) was also designed and listed in Table 1.

According to the above ratio, DNTF, NC, GAP, TDI and T-12 were all dissolved in acetone using VOSHIN-650W ultrasonic cell pulverizer (produced by Wuxi Woxin Instrument Co., Ltd., Wuxi, China). After the components were completely dissolved, the solid residue was filtered by suction filtration using a filter membrane with a diameter of 0.1 mm to prepare a transparent explosive ink.

### 2.3. Surface and Rheological Properties Test of Explosive Ink

Surface tension of the explosive ink was measured by QBZY automatic surface tension meter produced by Shanghai Fangrui Instrument Co., Ltd. (Shanghai, China). Viscosity of the explosive ink was tested by a LVDV-1 viscometer provided by Shanghai Fangrui Instrument Co., Ltd. (Shanghai, China) with the shear rate set as 60 s^−1^ and a temperature of 25 °C. In addition, density of the explosive ink was characterized by a MZ-220SD electronic densitometer supplied by Shenzhen Lidaxin Instrument Co., Ltd. (Shenzhen, China).

### 2.4. Inkjet Printing of Energetic Composites

The explosive ink was printed on the specimen under an optimized conditions by an inkjet printing device (self-assembled by North University of China). The air pressure, circulation, pulse and height of the piezoelectric nozzle were 0.05 MPa, 5.0 ms, 0.3 ms and 5 mm, respectively. The temperature of heating platform was 45 °C, and the writing speed was 50 mm·s^−1^. The inkjet printed sample was rectangular parallelepiped, length, width and thickness were 4.5 cm, 1.3 cm and 1.5 mm, respectively. Finally, the inkjet printed sample was placed in a water bath oven and cured at a temperature of 60 °C for 3–7 days.

### 2.5. Characterization and Testing of Inkjet Printed Samples

The surface and inner structure as well as density of DNTF-based inkjet printing samples were observed by Mira 3 TESCAN scanning electron microscope (SEM) and MZ-220SD electronic densimeter, respectively. In addition, a DX-2700 X-ray diffractometer (XRD, Dandong HaoYuan Instrument Co., Ltd., Dandong, China) was utilized to test the crystal type of main explosive when the starting, progressive and ending angles were 5°, 0.03°and 50°, respectively.

The thermal properties of raw DNTF and DNTF-based composites were characterized by a Q20P high differential scanning calorimeter (PDSC, TA Instruments, NewCastle, USA) with the processing conditions listed as follows: heating rates set as 5 K·min^−1^, 10 K·min^−1^, 15 K·min^−1^ and 20 K·min^−1^ successively and pressure set as 2 MPa by filling N_2_ atmosphere at a flow rate of 30 mL·min^−1^. 

**Figure 1:**
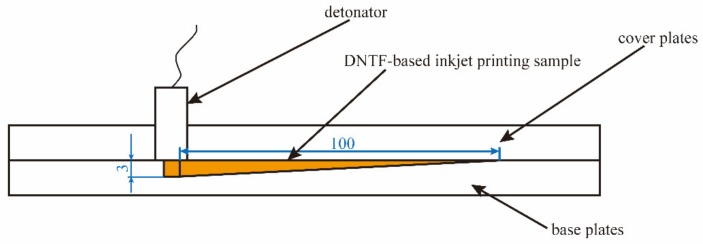
Schematic diagram of critical detonation thickness test for micro charge.

The critical detonation thickness of micro charge was tested according to the method in literature. Firstly, the explosive ink was printed layer by layer into a wedge groove with 1 mm width, whose specific size were shown in the schematic diagram (Figure 1). Secondly, the cover plate, base plate and detonator were assembled according to Figure 1. The micro charge in the wedge groove was detonated from the thick end and the extinction point could be observed easily. Finally, the critical detonation thickness of DNTF-based inkjet printing samples was calculated using the extinction length.

**Figure 2:**
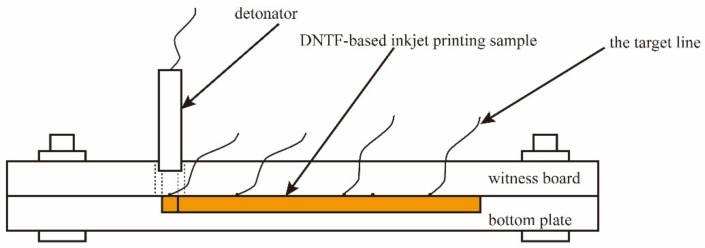
Detonation velocity test schematic diagram of micro charge.

The detonation velocity of micro charge was tested by means of the ionization probes method, which was also described in the literature. The experimental arrangements are shown in Figure 2. The linear micro charge was initiated by an electric detonator firstly, and then the time across the distance between each two probes was recorded. The detonation velocity can be calculated in terms of the distance and time.

The mechanical behavior of DNTF-based inkjet printing samples was captured by using a static loading model of Nano Indenter G200 with a loading stress of 0 to 500 mN and a wedge-shaped tip. The elastic modulus and hardness of the molded samples were extracted by using Agilent NanoSuit, and each sample was tested five times to obtain an average value.

## 3. Results and Discussion

### 3.1. Printability of Explosive Ink

For the piezoelectric nozzle, the printability of explosive ink can be estimated by a dimensionless number Z, which is defined as Equation (1) [30,31,32,33]:

(1) $$Z=\frac{{\left(\alpha \rho \gamma \right)}^{\frac{1}{2}}}{\eta }$$

where α represents the diameter of the printing orifice, ρ, γ and η represent the density, surface tension and viscosity of the ink, respectively. 

Inkjet printing is a fluid motion process, and the hydrodynamic properties of explosive inks determine whether they can form stable ink droplets and fall off from the nozzle. The hydrodynamic properties mainly involve the viscosity, density and surface tension of explosive inks. Fromm’s [31] research shows that in the ink deposition printing ink forming, in order for the ink droplets to drip out from the nozzle smoothly, the Z value of the explosive ink must be greater than 2. By a series of physical property tests, the surface tension, viscosity and density of explosive ink are determined as 24.656 mN·m^−1^, 1.4 mPa·s and 840 kg·m^−3^, respectively. The Z value of the explosive ink can be calculated using Equation (1) to be 32.51. Therefore, the formulated explosive ink can meet the requirements of inkjet printing.

### 3.2. Printing and Molding Mechanism of Energetic Composites

**Figure 3:**
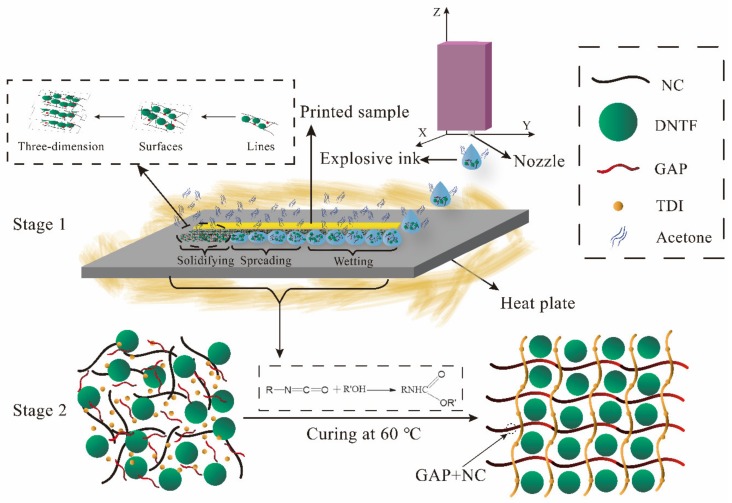
Diagrammatic sketch of inkjet printing (Stage 1) and curing reaction (Stage 2) of 3,4-dinitrofurazanofuroxan (DNTF)-based explosive ink.

The molding process of the explosive ink can be divided into two stages as shown in Figure 3: the inkjet printing molding stage and the post-curing reaction stage. (1) In stage 1, the micro-scale ink droplets generated by the piezoelectric nozzle are dropped onto the designated location on demand using a high-precision 3D motion platform. Subsequently, the ink drops are wetting, spreading and solidifying on the substrate. From the moment the ink droplets formed, the solvent begins to volatilize. Due to the supersaturation, the solutes (explosive and binder) are crystallized gradually, and eventually the ink droplets can completely turn into a solid phase to form a printed sample. By optimizing the inkjet printing parameters, the energetic composite can be formed from point to line, to 2D area and to 3D body. (2) Stage 2 refers to the cross-linking reaction between the polymer group and the curing agent after the preliminary curing of the explosive ink, so that the mechanical properties of the molded sample can be enhanced.

The bonding system of the explosive ink formulation is designed in this thesis mainly includes GAP, NC and TDI. Although GAP and NC are both linear chain polymers, they are showing different states at the room temperature and atmospheric pressure. The solid NC and liquid GAP play different roles in the inkjet printing stage. With the evaporation of acetone solvent, NC can be quickly precipitated and solidified on the designated location. However, the liquid polymer GAP cannot change into solid state alone, but it can absorb into the cellulose molecule to solidify. Therefore, NC is an indispensable substance in the preliminary forming stage, which ensure ink jet printing precision and molding effect. 

Owing to the linear chain polymer of GAP and NC, the interaction between different components are mainly the weak force, such as van der Waals forces and intermolecular hydrogen bonds, which result in the poor mechanical properties of the GAP/NC/DNTF printing sample. As is known, TDI is an isocyanate compound, which has been used as curing agent in GAP system. In this experiment, TDI plays an important role in the post-cure reaction stage. The isocyanate in TDI can cross-link with the hydroxyl groups in GAP and NC to form a covalent bond. The transformation of the polymer chain structure into a high-strength three-dimensional network structure enables the energetic composite to have better mechanical properties [34,35,36].

### 3.3. SEM Characterization

**Figure 4:**
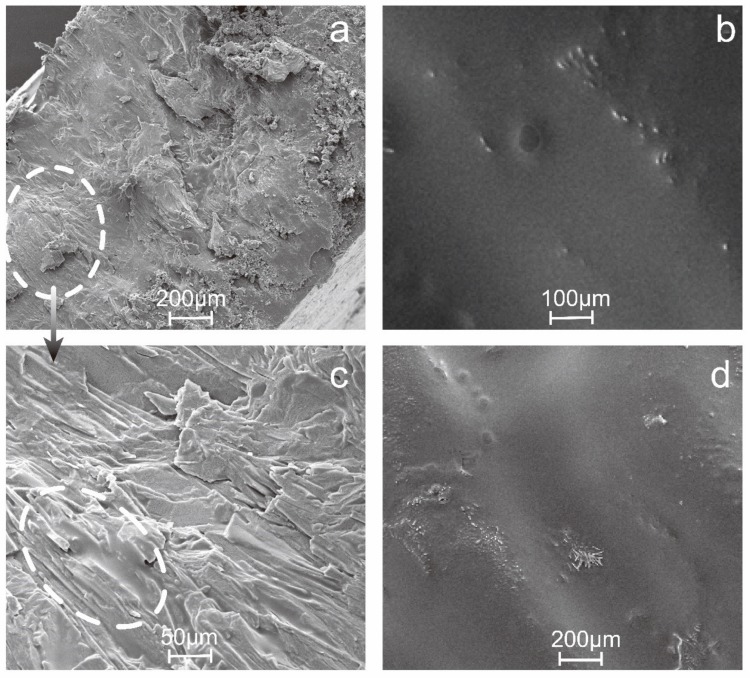
Scanning Electron Microscope (SEM) images of GNT printed samples at different magnification: (**a**,**c**) cutting cross sectional view; (**b**,**d**) surface view.

The surface and cross-sectional microstructure of DNTF-based inkjet printing samples are obtained by SEM, and the results are shown in Figure 4. It can be clearly seen in Figure 4a,b that GAP, NC and DNTF has been integrated as a whole, no particles and pores are observed in the inner of the printed samples, showing the dense microstructure. In Figure 4c, the layered microstructure can be found distinctly in a larger magnification, in which the layers connect tightly with each other and the thickness of them is less than 10 μm. It was the embodiment of the layer by layer assembly printing process. In Figure 4b,d, it can be seen that the printed sample has a dense and smooth surface, showing a good molding effect.

### 3.4. Theoretical and Measured Density

The density of printed samples of the GNT ink and GN ink is tested by an electron densitometer. Their theoretical density is calculated by Equation (2) [37]. The results are shown in Table 2.

(2) $$\rho_{e}=\frac{\sum m_{i}}{\sum \frac{m_{i}}{\rho_{i}}}$$

where $\rho_{e}$ is the theoretical density of the explosive, g·cm^−3^; $m_{i}$ is the mass of the i component, g; $\rho_{i}$ is the density of the i component, g·cm^−3^.

As can be seen in Table 1, although there is a little difference in formulation of GN and GNT ink, the printed samples have the same theoretical density (TMD), which is calculated as 1.857 g·cm^−3^. The measured density of the GNT and GN printed sample is determined as 1.773 g·cm^−3^ and 1.763 g·cm^−3^, respectively, the ratio of measured density and theoretical density for GNT and GN printed sample is 95.5% and 95.0%, respectively. Thus, it can be seen that DNTF based printed samples with high density can be easily obtained by such printing process. It is mainly because DNTF is easy to form layered crystals during recrystallization. Compared with other crystal shape, the layered crystals are easier to construct a dense micro charge without gaps by layer-by-layer assembling. This argument can be clearly seen in the SEM images of Figure 4. By contrast of the density of the GNT and GN printed samples, it can be found that the density of micro charge increased about 0.01 g·cm^−3^ by the polymer cross-linking reaction. The covalent bond increases the intermolecular force and further increases the density of DNTF-based inkjet printing samples.

### 3.5. XRD Patterns

**Figure 5:**
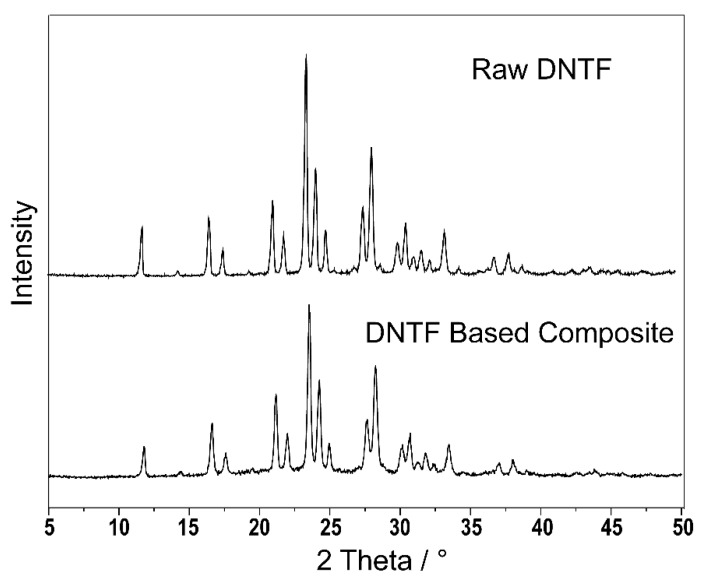
X-ray Diffractometer (XRD) test pattern of DNTF-based inkjet printing samples.

As is shown in Figure 5, the diffraction angle of DNTF based inkjet printing samples is similar to that of raw DNTF, indicating that the crystal form of DNTF does not change in the printing process.

### 3.6. Mechanical Performance

**Figure 6:**
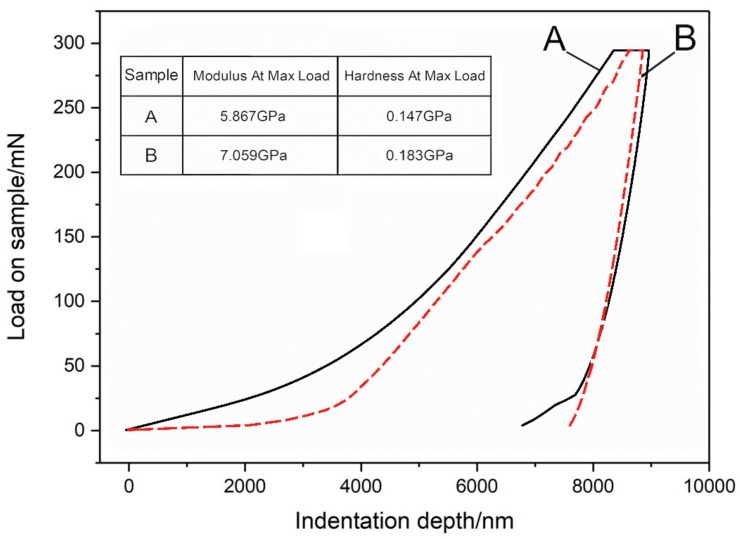
The load-displacement curve of DNTF-based inkjet printing samples: (**A**) a GN ink inkjet printing sample; (**B**) a GNT ink inkjet printing sample. The modulus and hardness values of the two samples at max load are tabled in the figure.

The mechanical properties of GNT and GN printed samples are tested by a Nanoindentation method and the results are shown in Figure 6. The curve in Figure 6 is a typical load-displacement curve [38] obtained after a load-unload cycle in the indentation experiment, from which the elastic modulus and hardness of the sample can be calculated. Elastic modulus is an index to measure the resistance of an object to elastic deformation, while hardness is the resistance to elastic deformation, plastic deformation or failure.

It can be seen from Figure 6 that, compared with the GN printed samples without curing agent, the load-displacement curve of the GNT printed samples with curing agent moves to the right, and the maximum indentation depth also become deeper. The elastic modulus and hardness of the printed samples can be calculated from the maximum load of the load-displacement curve, the maximum indentation depth, the remaining displacement after complete unloading, and the slope at the top of the unloading curve. As is shown in the table in Figure 6, by such curing technology, the elastic modulus and hardness of the printed samples increased by 1.192 GPa (20.3%) and 0.036 GPa (24.5%), respectively. The growth rate has exceeded 20%. Therefore, by the curing of polymer in the printed sample, the strength of printed energetic sample is increased evidently [39,40].

### 3.7. PDSC Curves Analysis

**Figure 7:**
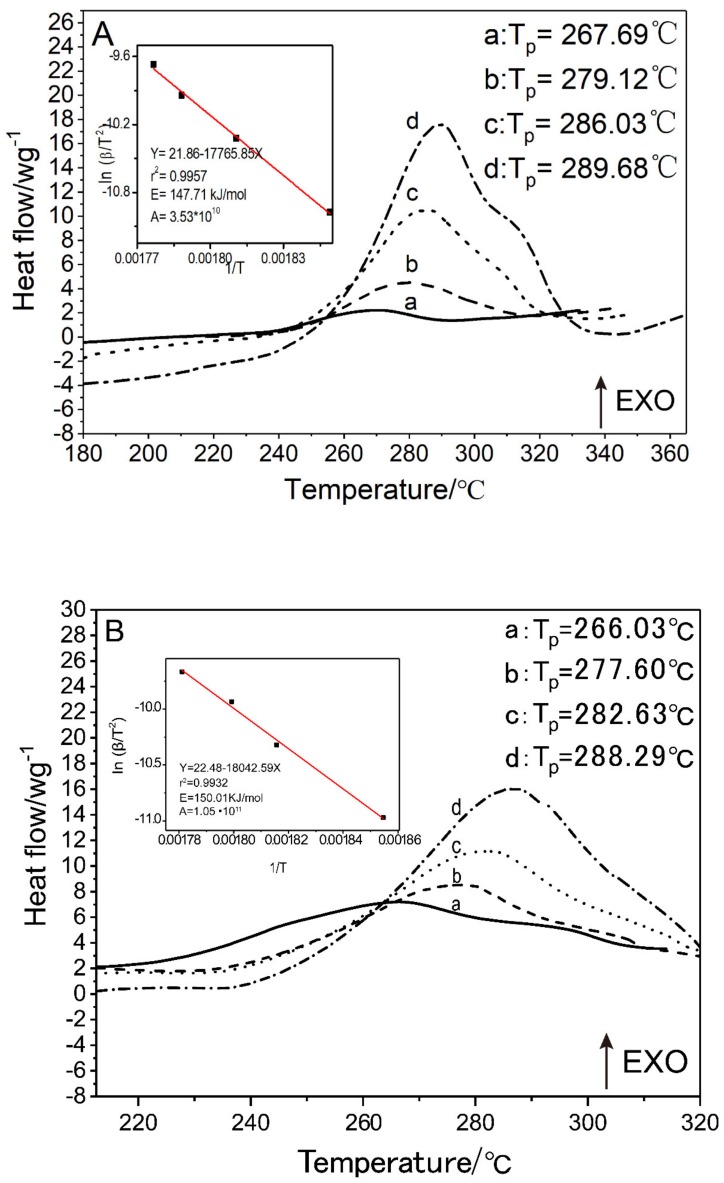
Q20P high differential scanning calorimeter (PDSC) curves at heating rate of 5 K·min^−1^ (a), 10 K·min^−1^ (b), 15 K·min^−1^ (c) and 20 K·min^−1^ (d) under the nitrogen atmosphere of 2 MPa: (**A**) raw DNTF samples; (**B**) GNT printed samples. The insets are Kissinger’s plots for raw DNTF samples and GNT printed samples, respectively.

Raw DNTF and GNT printed samples are characterized by PDSC at heating rates of 5 K·min^−1^, 10 K·min^−1^, 15 K·min^−1^ and 20 K·min^−1^, respectively. The results are shown in Figure 7.

The decomposition peak temperature of the GNT printed samples at different heating rates is slightly lower than that of the raw material DNTF, which indicates that the bonding system (GAP and NC) has little effect on the thermal decomposition of DNTF.

Activation energy of raw DNTF and GNT samples are calculated by the Kissinger Equation (3) [41] with the observed exothermic peaks of DNTF, which are 147.11 kJ·mol^−1^ and 150.01 kJ·mol^−1^, respectively. Compared with the raw material DNTF, the activation energy of the GNT samples have increased, which indicates that the thermal sensitivity of the energetic compound has decreased, and it is more difficult to decompose when heated, making it more stable than the raw material DNTF.

(3) $$ln\frac{\beta_{i}}{T_{pi}^{2}}=ln\frac{AR}{E}-\frac{E}{RT_{pi}}$$

where $\beta_{i}$ is the heating rate, K·min^−1^; $T_{pi}$ is the exothermic peak at heating rate of $\beta_{i}$, K; E is the apparent activation energy, kJ·mol^−1^; A is the pre-exponential factor; R is the gas constant (8.314 J·mol^−1^·K^−1^).

### 3.8. Critical Thickness of Detonation

**Figure 8:**
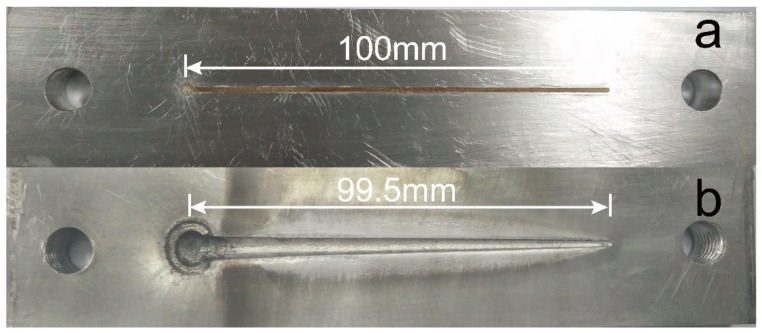
Specimen images of the critical detonation thickness test for GTN printed samples: (**a**) before detonation; (**b**) after detonation.

The critical thickness of detonation is a crucial indicator to reflect the detonation ability at micro-charge size. The specimen photos before and after the test are shown in Figure 8.

From Figure 8, it can be seen that the DNTF based energetic composites have been printed into the wedge-shaped groove with 1mm width. After initiation, the liner explosive is detonated and expands the groove size. From left to right, the groove width decreases slightly with the reduction of wedge charge thickness. If the charge thickness is lower than the critical detonation thickness, the detonation will be extinct. In Figure 8b, the detonation distance is determined as 99.5 mm. It is calculated that the critical thickness of detonation of GNT printed samples is 0.015 mm by the reported method [42]. Therefore, GNT printed samples can be stably detonated at micron charge and has a good application prospect in the micro-initiation system.

### 3.9. Detonation Velocity

**Figure 9:**
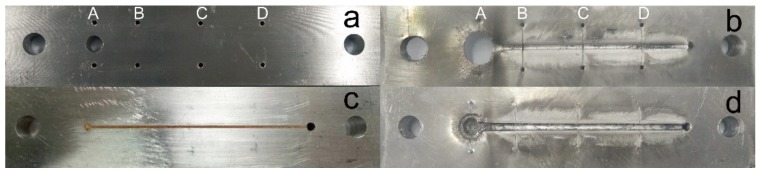
Detonation velocity test specimen images of GNT printed samples by means of the ionization probes method: (**a**,**c**) optical image of cover plate and GNT explosive-deposited plate before detonation; (**b**,**d**) optical image of cover plate and GNT explosive-deposited plate after detonation.

The detonation velocity of the sample is tested by means of the ionization probes method, and the test specimen photos are shown in Figure 9.

It can be seen from Figure 9, the groove width is evenly broadened from 1mm to about 3 mm, indicating the high charge homogeneity. The liner micro-charge is initiated at A and detonated completely from left to right in groove. The stage of A–B presents a trend of detonation growth, in which the detonation trace is unstable, while the stages of B–C and C–D showing stable detonation processes. Thus, B–C and C–D are utilized to calculate the detonation velocity. The experimental data in Table 3 shows that the average detonation velocity of the GNT printed samples is as high as 8686 m·s^−1^, which also makes a new height in the field of micro-scale detonation. The theoretical detonation velocity of GNT printed samples is also calculated as 8891 m·s^−1^ using Equation (4).

(4) $$\begin{array}{c} D_{max}=\sum D_{i}a_{vi}\\ a_{vi}=\rho_{max}\frac{g_{i}}{\rho_{imax}}\\ D=\frac{D_{max}}{4}+\frac{3}{4}\frac{D_{max}}{\rho_{max}}\rho_{0} \end{array}$$

where $D_{max}$ is the theoretical detonation velocity; $D_{i}$ is the theoretical detonation velocity in an ingredient; $a_{vi}$ is the volume fraction of the $i^{th}$ ingredient; $\rho_{max}$ is the theoretical density of the explosive compound; $g_{i}$ is the ingredient quality; $\rho_{imax}$ is the theoretical density of the $i^{th}$ ingredient; $\rho_{0}$ is the measured density; D is the theoretical detonation velocity when $\rho_{0}$ is the measured density.

## 4. Conclusions

In this paper, the inkjet printing micro-fabrication technology and polymer cross-linking technology are combined to successfully print a GAP/NC/DNTF micro-scale energetic composite sample with high strength, high detonation speed and small critical dimension. Compared with the direct writing process, inkjet printing technology has a higher precision, so it is easier to realize the molding of microstructure and improve the quality consistency of the samples. Herein, the inkjet printing process realizes the orderly assembly of the explosive/binder energetic composite. The obtained printed sample has a smooth surface and a dense internal microstructure, and the single layer printing thickness is less than 10 μm. The post-cure reaction of GAP/NC and TDI further improved the mechanical properties and charge density of the micro-charge. The elastic modulus and hardness of the micro-charge increased by more than 20% and the molding density increased from 1.763 to 1.773 g·cm^−3^ by curing technology. The obtained micro-charge has excellent micro-scale detonation ability: the critical detonation thickness and detonation velocity can reach 0.015 mm and 8686 m·s^−1^, respectively. This paper provides a new research idea for the design and preparation of micro-charge with high-strength and high-detonation.

## Figures and Tables

**Table 1:** The formulation of GNT and GN ink.

Formulations	Main Explosive (wt.%)	GAP (wt.%)	Cellulose Polymer (wt.%)	TDI (wt.%)	T-12 (wt.%)	Acetone (wt.%)
GNT ink	14.98	1.33	0.33	0.08	0.04	83.24
GN ink	15.00	1.33	0.33	-	-	83.34

**Table 2:** Theoretical density and measured density of 3,4-dinitrofurazanofuroxan (DNTF)-based printed samples.

Experiment No.	Density/g·cm^−3^			
	GNT ink-1	GNT ink-2	GN ink-1	GN ink-2
Experiment 1	1.769	1.776	1.759	1.764
Experiment 2	1.778	1.773	1.766	1.761
Experiment 3	1.773	1.768	1.761	1.768
Average	1.773	1.772	1.762	1.764
Theoretical density	1.857		1.857	

**Table 3:** Detonation velocity of DNTF-based inkjet printing sample.

Test Section	B–C	C–D
Distance/mm	30	30
Time/ns	3361	3430
Detonation speed/m·s^−1^	8626	8746
Average detonation speed/m·s^−1^	8686	
Theoretical detonation/m·s^−1^	8891	
